# Supplementary material for: Genome-wide association studies of metabolites in Finnish men identify disease-relevant loci
Source: Nat Commun. 2022 Mar 28;13:1644. doi: 10.1038/s41467-022-29143-5 (PMC8960770; doi:10.1038/s41467-022-29143-5)
Supplement: Supplementary file 3 — Description of Additional Supplementary Files [file 41467_2022_29143_MOESM3_ESM.pdf]

Description of Additional Supplementary Files

File Name: Supplementary Data 1
Description: List of the 1,544 metabolites in the METSIM Metabolon metabolomics study

File Name: Supplementary Data 2
Description: List of 83 metabolite genetic associations whose effect size ratios exceed 1.2 between the association models with and without BMI adjustment

File Name: Supplementary Data 3
Description: All 2,030 significant single-variant association results identified in stepwise conditional analysis at P< 7.2×10-11 Supplementary Data 4: 99 index variants that explain ≥10% of the phenotypic variance for 91 metabolites

File Name: Supplementary Data 4
Description: 99 index variants that explain ≥10% of the phenotypic variance for 91 metabolites

File Name: Supplementary Data 5
Description: 1,952 significant association signals with signal posterior inclusion probability≥0.95 in Bayesian statistical fine-mapping analysis

File Name: Supplementary Data 6
Description: Previous metabolite genetic associations with P< 5×10-8 or stronger study-specific p-value threshold at 8,502 genetic variants in 381 publications

File Name: Supplementary Data 7
Description: 277 putative causal genes nominated in the knowledge-based approach

File Name: Supplementary Data 8
Description: Comparison of gene nominations for 138 association signals between fine mapping and the knowledgebased approach

File Name: Supplementary Data 9
Description: List of the 980 diseases and disease-related dichotomous traits with FINEMAP fine-mapping results in the FinnGen release 4

File Name: Supplementary Data 10
Description: 946 significant genetic colocalizations between 105 FinnGen release 4 disease traits and 248 metabolites (RCP≥0.5)
