# Supplementary material for: Genome-wide association studies of metabolites in Finnish men identify disease-relevant loci
Source: Nat Commun. 2022 Mar 28;13:1644. doi: 10.1038/s41467-022-29143-5 (PMC8960770; doi:10.1038/s41467-022-29143-5)
Supplement: Supplementary file 14 — Reporting Summary [file 41467_2022_29143_MOESM14_ESM.pdf]

Corresponding author(s): Michael Boehnke, Markku Laakso, Eric B Fauman

Last updated by author(s): Jan 17, 2022

Reporting Summary

Nature Portfolio wishes to improve the reproducibility of the work that we publish. This form provides structure for consistency and transparency in reporting. For further information on Nature Portfolio policies, see our Editorial Policies and the Editorial Policy Checklist.

Statistics

For all statistical analyses, confirm that the following items are present in the figure legend, table legend, main text, or Methods section.

n/a	Confirmed	
☐	☒	The exact sample size (*n*) for each experimental group/condition, given as a discrete number and unit of measurement
☐	☒	A statement on whether measurements were taken from distinct samples or whether the same sample was measured repeatedly
☐	☒	The statistical test(s) used AND whether they are one- or two-sided<br>*Only common tests should be described solely by name; describe more complex techniques in the Methods section.*
☐	☒	A description of all covariates tested
☐	☒	A description of any assumptions or corrections, such as tests of normality and adjustment for multiple comparisons
☐	☒	A full description of the statistical parameters including central tendency (e.g. means) or other basic estimates (e.g. regression coefficient) AND variation (e.g. standard deviation) or associated estimates of uncertainty (e.g. confidence intervals)
☐	☒	For null hypothesis testing, the test statistic (e.g. *F*, *t*, *r*) with confidence intervals, effect sizes, degrees of freedom and *P* value noted<br>*Give P values as exact values whenever suitable.*
☐	☒	For Bayesian analysis, information on the choice of priors and Markov chain Monte Carlo settings
☒	☐	For hierarchical and complex designs, identification of the appropriate level for tests and full reporting of outcomes
☐	☒	Estimates of effect sizes (e.g. Cohen's *d*, Pearson's *r*), indicating how they were calculated

Our web collection on statistics for biologists contains articles on many of the points above.

Software and code

Policy information about availability of computer code

Data collection	No software was used.
Data analysis	We used the following open source softwares:<br>Picard v2.4.1: generate quality control statistics for exome and genome sequencing data<br>Samtools v1.3.157: generate quality control statistics for exome and genome sequencing data<br>VerifyBamID v1.1.3: generate quality control statistics for exome and genome sequencing data<br>GATK v3.5: call genetic variants in exome and genome sequencing data<br>KING v2.2.1: estimate kinship among individuals from genotype data<br>Beagle v4.1: haplotype estimation<br>Minimac v4: genotype imputation<br>Ensembl Variant Effect Predictor (VEP) version 99: genetic variant functional annotation<br>EPACTS v3.2.6: genetic association tests<br>DAP-g v1.0: multi-SNP statistical fine-mapping<br>TORUS v1.0: QTL Discovery utilizing Genomic Annotations<br>fastENLOC v1.0: Bayesian colocalization analysis between metabolite and disease genetic associations<br>FuzzyMatch v2: a pair of terms distance score calculator<br>R v4.0: data processing, computing and plotting

For manuscripts utilizing custom algorithms or software that are central to the research but not yet described in published literature, software must be made available to editors and reviewers. We strongly encourage code deposition in a community repository (e.g. GitHub). See the Nature Portfolio guidelines for submitting code & software for further information.

Data

Policy information about availability of data

All manuscripts must include a data availability statement. This statement should provide the following information, where applicable:

- Accession codes, unique identifiers, or web links for publicly available datasets
- A description of any restrictions on data availability
- For clinical datasets or third party data, please ensure that the statement adheres to our policy

NHGRI-EBI GWAS catalog: https://www.ebi.ac.uk/gwas/. Human Metabolic Individuality: http://www.metabolomix.com/list-of-all-published-gwas-with-metabolomics/. OMIM: https://omim.org/. KEGG: https://www.kegg.jp/. HMDB: https://hmdb.ca. GTEx portal: https://gtexportal.org/home/. NCBI refSeq Gene: https://www.ncbi.nlm.nih.gov/refseq/rsg/. Entrez Gene: https://www.ncbi.nlm.nih.gov/gene. UniProt: https://www.uniprot.org. Gene Ontology: http://geneontology.org. dbNSFP: https://sites.google.com/site/jpopgen/dbNSFP. FinnGen genome-wide summary statistics and Bayesian statistical fine-mapping results are available at https://r4.finngen.fi. Full summary statistics from the genome-wide association studies of the 1,391 plasma metabolites are available at https://pheweb.org/metsim-metab/. METSIM individual-level data are not publicly available due to privacy restrictions on personal data. The METSIM exome sequencing and genotyping array data will be accessible through dbGaP (https://www.ncbi.nlm.nih.gov/gap/) with accession numbers phs000752 and phs000919, respectively. The METSIM WGS dataset used in this manuscript (n=5,949) is a subset of the full METSIM WGS data which will be deposited into dbGaP upon completion, expected in early 2022. The METSIM metabolomics dataset (n=6,490) is a subset of the full METSIM metabolomics data which will be deposited into dbGaP upon completion, expected in March-May 2022. As part of data deposit in dbGAP, we will include ID lists corresponding to the individuals included in this paper. Until sequence and metabolomics data are available from dbGaP, we will provide access to the data for this paper under a Data Use Agreement upon reasonable request to Dr. Michael Boehnke (boehnke@umich.edu).

Field-specific reporting

Please select the one below that is the best fit for your research. If you are not sure, read the appropriate sections before making your selection.

[x] Life sciences [ ] Behavioural & social sciences [ ] Ecological, evolutionary & environmental sciences

For a reference copy of the document with all sections, see nature.com/documents/nr-reporting-summary-flat.pdf

Life sciences study design

All studies must disclose on these points even when the disclosure is negative.

Sample size	METSIM is a study of 10,197 Finnish men from Kuopio in the late settlement region of northeast Finland. For this metabolomics study, no statistical method was applied to predetermine the study sample size. We used all 6,490 METSIM participants with Metabolon metabolomics data available.
Data exclusions	We excluded individuals either diagnosed with diabetes or taking diabetes medications, lacked array genotypes (n=68) or body mass index (BMI) measurement, had sex mismatch, were non-Finnish, or were outliers in genetic principal component analysis (PCA). We limited analysis to the 1,391 metabolites successfully measured on ≥500 METSIM participants.
Replication	We replicated 1,727 (85% of our findings) metabolite genetic associations that were previously reported by independent studies. Details are provided in the manuscript.
Randomization	This is an observational study. Randomization was not applicable.
Blinding	This is an observational study. Blinding was not applicable.

Reporting for specific materials, systems and methods

We require information from authors about some types of materials, experimental systems and methods used in many studies. Here, indicate whether each material, system or method listed is relevant to your study. If you are not sure if a list item applies to your research, read the appropriate section before selecting a response.

Materials & experimental systems

n/a	Involved in the study	
[x]	[ ]	Antibodies
[x]	[ ]	Eukaryotic cell lines
[x]	[ ]	Palaeontology and archaeology
[x]	[ ]	Animals and other organisms
[ ]	[x]	Human research participants
[x]	[ ]	Clinical data
[x]	[ ]	Dual use research of concern

Methods

n/a	Involved in the study	
[x]	[ ]	ChIP-seq
[x]	[ ]	Flow cytometry
[x]	[ ]	MRI-based neuroimaging

Human research participants

Policy information about studies involving human research participants

Population characteristics	METSIM is a study of 10,197 Finnish men aged 45 to 74 (median 58) years at baseline from Kuopio in the late settlement region of northeast Finland. For this metabolomics study, we randomly selected 6,490 of the 8,777 METSIM participants who at baseline were neither diagnosed with diabetes nor taking diabetes medications. The demographic characteristics of the cohort are fully detailed in Supplementary Table 3.
Recruitment	METSIM is a prospective study to investigate the genetic and non-genetic factors for diabetes and cardiovascular diseases. The participants were recruited from Kuopio in the late settlement region of northeast Finland. Baseline visits were undertaken from 2005-2010. Follow-up visits started in 2010. At both visits, participants provided demographic, diet, exercise, disease, and medication history information, and underwent laboratory measurements, including oral glucose tolerance test, after ≥10-hour overnight fast. Morbidity, mortality, and drug treatment information was periodically updated for participants who consented to use of their hospital admission, drug reimbursement, and prescription records in Finnish national registries.
Ethics oversight	This study was approved by the Ethics Committee at the University of Eastern Finland and the Institutional Review Board at the University of Michigan.

Note that full information on the approval of the study protocol must also be provided in the manuscript.
